# Prediction model for thyrotoxic atrial fibrillation: a retrospective study

**DOI:** 10.1186/s12902-021-00809-3

**Published:** 2021-07-11

**Authors:** Daria Aleksandrovna Ponomartseva, Ilia Vladislavovich Derevitskii, Sergey Valerevich Kovalchuk, Alina Yurevna Babenko

**Affiliations:** 1 Almazov National Medical Research Centre, Institute of Endocrinology, 15 Parkhomenko street, St. Petersburg, 194156 Russia; 2 ITMO University, 9 Lomonosova street, Saint Petersburg, 191002 Russia

**Keywords:** Thyrotoxicosis, Atrial fibrillation, Thyrotoxic atrial fibrillation, Graves’ disease, Prediction model, Machine learning

## Abstract

**Background:**

Thyrotoxic atrial fibrillation (TAF) is a recognized significant complication of hyperthyroidism. Early identification of the individuals predisposed to TAF would improve thyrotoxic patients’ management. However, to our knowledge, an instrument that establishes an individual risk of the condition is unavailable. Therefore, the aim of this study is to build a TAF prediction model and rank TAF predictors in order of importance using machine learning techniques.

**Methods:**

In this retrospective study, we have investigated 36 demographic and clinical features for 420 patients with overt hyperthyroidism, 30% of which had TAF. At first, the association of these features with TAF was evaluated by classical statistical methods. Then, we developed several TAF prediction models with eight different machine learning classifiers and compared them by performance metrics. The models included ten features that were selected based on their clinical effectuality and importance for model output. Finally, we ranked TAF predictors, elicited from the optimal final model, by the machine learning tehniques.

**Results:**

The best performance metrics prediction model was built with the extreme gradient boosting classifier. It had the reasonable accuracy of 84% and AUROC of 0.89 on the test set. The model confirmed such well-known TAF risk factors as age, sex, hyperthyroidism duration, heart rate and some concomitant cardiovascular diseases (arterial hypertension and conjestive heart rate). We also identified premature atrial contraction and premature ventricular contraction as new TAF predictors. The top five TAF predictors, elicited from the model, included (in order of importance) PAC, PVC, hyperthyroidism duration, heart rate during hyperthyroidism and age.

**Conclusions:**

We developed a machine learning model for TAF prediction. It seems to be the first available analytical tool for TAF risk assessment. In addition, we defined five most important TAF predictors, including premature atrial contraction and premature ventricular contraction as the new ones. These results have contributed to TAF prediction investigation and may serve as a basis for further research focused on TAF prediction improvement and facilitation of thyrotoxic patients’ management.

## Background

Hyperthyroidism is associated with an increase in both total and cardiovascular mortality [1]. The majority of patients with hyperthyroidism are working age individuals. Consequently, its negative social impact is highly significant [2].

Atrial fibrillation (AF) is the most common severe complication of hyperthyroidism. It is known to provoke both thromboembolic events and heart failure and increase mortality [3]. The thyrothoxic AF (TAF) incidence is as follows: 7–8% among middle-aged patients, 10–20% in seniors and 20–35% for those having coronary heart disease or valvular disease [4–6]. Hence, TAF prevention is a crucial problem.

To date, a fairly large number of TAF predictors have been identified. Advanced age [4, 5, 7–14], concomitant cardiovascular diseases [4, 5, 7, 15] and male gender [4, 5, 8, 14] are the most widely acknowledged. Prolonged duration of hyperthyroidism [14] and increased heart rate [12, 14] are the less investigated TAF predictors. Nonimmune genesis of thyrotoxicosis [5, 16] is also shown to be associated with increased TAF prevalence. But it is considered to be caused by the old age of these patients [17].

Moreover, few studies mentioned new TAF risk factors listed below. They are, undoubtedly, less explored and need to be confirmed. The obesity, presence of chronic kidney disease, proteinuria, increased levels of hepatic transaminases and C-reactive protein are shown to raise TAF risk [12, 18]. Conversely, the use of beta-blockers, angiotensin-converting enzyme inhibitors or antiarrhythmic drugs before hyperthyroidism is associated with a lower TAF frequency [9, 12, 18].

The findings regarding thyroid hormones level have been controversial. Generally, when investigating overt hyperthyroidism, an association of free triiodothyronine (fT3) or free thyroxine (fT4) level with TAF frequency [4, 13, 14, 19], has not been revealed. By contrast, some researchers have demonstrated that fT3 and fT4, [9] or fT4 exclusively [18], have been higher among patients with TAF.

Therefore, to date, many TAF predictors are known, but the information appears to be insufficient and controversial. In addition, no TAF prediction tool has been developed. To the best of our knowledge, we did this for the first time.

Since machine learning can improve the accuracy of the prediction, and its application in the medical field has yielded promising results [20–23], we used it to develop our model. Machine learning is a data-driven approach that can identify nonlinear associations and complex interactions between variables without the need to pre-specify these relationships a priori [24]. Thereby, in modeling risk, the machine learning is doing more than merely approximating physician skills but finding novel relationships not readily apparent to human beings [21]. Starting with patient-level observations, algorithms sift through vast numbers of variables, looking for combinations that reliably predict outcomes [20]. All this makes machine learning an excellent method for prediction instruments construction. Thus, the purpose of this study was to build a TAF prediction model and rank TAF predictors in order of importance using machine learning techniques.

## Methods

### Participants

This is a retrospective observational study of 420 patients with overt hyperthyroidism, including 127 TAF cases.

All participants had undergone or were undergoing outpatient or inpatient hyperthyroidism treatment in Almazov National Medical Research Centre or Pavlov First Saint Petersburg State Medical University between December 2000 and December 2019. Firstly, to select the eligible subjects, hyperthyroid patient medical records were examined. Secondly, to document a patient case history a single office visit was arranged. Finally, tracing the disease dynamics was fulfilled by phone. Local Ethics Committee approval was obtained. And, prior to the research, all participants had signed the informed consent form.

The participants were recruited in accordance with the criteria listed below.

Entry criteria:

1. Men and women with a history of overt hyperthyroidism, associated with Graves’ disease (GD), toxic adenoma (TA) or multinodular toxic goiter (MTG).
2. Age between 18 and 80 years.

Exclusion criteria:

1. Subclinical hyperthyroidism (without the period of overt hyperthyroidism).
2. A history of AF developed before the onset of hyperthyroidism.
3. Concomitant diseases:, severe obstructive lung diseases, severe blood disorders, severe organ failure.
4. Chronic intoxication (alcohol, narcomania, toxicomania).
5. Pregnancy at the time of hyperthyroidism.

### Data collection and ascertainment of clinical features

Project data were collected retrospectively, from the in-patient and out-patient medical records (including electronic medical records), face-to-face and telephone patient inquiries.

The dataset contained 36 study variables classified into six categories: demographic data, characteristics of hyperthyroidism course, cardiological status before and during hyperthyroidism, some metabolic parameters and blood tests, smoking status and heart rate-reducing therapy (Table 1). The variables were selected based on recognized or possible associations with TAF.

**Table 1:** Study variables

Categories of the variables (number of the variables in the group)	Study variables
**Demographic data (2)**	• Sex • Age at the onset of hyperthyroidism
**Characteristics of hyperthyroidism course (11)**	• Thyroid function: ▪ thyroid-stimulating hormone level ▪ free triiodthyronine level ▪ free tetraiodthyronine level • Thyroid-stimulating hormone receptors antibodies level (only in subjects with Graves’ disease) • Hyperthyroidism duration (for patients with AF - before AF development), months • Subclinical hyperthyroidism duration (more/less than year) • The periods of hypothyroidism (absence/presence) • The relapses of hyperthyroidism (the number of relapses) • Weight loss at the onset of hyperthyroidism (during first 1–6 months) • Hyperthyroidism genesis (Graves’ disease, toxic adenoma, multinodular toxic goiter) • Extrathyroidal Graves’ disease manifestations (ophthalmopathy, pretibial myxedema)
**Metabolic parameters, smoking status and blood tests during hyperthyroidism (11)**	• Body mass index • Carbohydrate metabolism disorders (diabetes mellitus, impaired fasting glucose, impaired glucose tolerance) • Lipid panel: ▪ Total cholesterol ▪ Triglycerides ▪ High density lipoproteins ▪ Low density lipoproteins • Smoking history • Potassium serum level • Hemoglobin level • Renal function: ▪ Serum creatinine concentration ▪ Estimated glomerular filtration rate
**Initial cardiovascular status (4)**	• Arterial hypertension (absence/presence, target ABP/ABP above target) • Coronary heart disease (absence/presence, a prior history of myocardial infarction) • Rhythm disorders (premature atrial and ventricular contraction, supraventricular and non-sustained ventricular tachycardia, wandering of atrial pacemaker) • Congestive heart failure (absence/presence)
**Cardiovascular status during hyperthyroidism** **(6)**	• Arterial hypertension (absence/presence, target ABP/ above target ABP) • Heart rate (recorded during physical examination, conducted in the period of overt hyperthyroidism before the therapy initiation), beat per minute • Rhythm disorders: ▪ premature atrial contraction ▪ premature ventricular contraction ▪ other: supraventricular and non-sustained ventricular tachycardia, wandering of atrial pacemaker • Congestive heart failure (absence/presence)
**Heart rate-reducing therapy (2)**	• Heart rate-reducing therapy before *hyperthyroidism* • Heart rate-reducing therapy during hyperthyroidism

*AF* atrial fibrillation; *ABP* arterial blood pressure

Thyroid status and other laboratory measurements were assessed at the time of the newly diagnosed hyperthyroidism, before thyrostatic drugs administration. Due to the distinction in reference intervals, thyroid hormones and thyroid-stimulating hormone (TSH) receptors antibodies values were evaluated as elevation above upper limit of normal (ULN).

Hyperthyroidism duration was established in months since the first clinical manifestations until euthyroid state was reached*.* Subclinical hyperthyroidism duration, the number of relapses and hypothyroidism periods were identified by repeated clinical thyroid status control.

The cardiovascular status was assessed before and during thyrotoxicosis. In TAF patients it was assessed prior to AF development. Initial cardiovascular status involved hypertension*,* coronary heart disease, cardiac arrhythmias and heart failure, diagnosed before hyperthyroidism development. Cardiovascular status during hyperthyroidism comprised the presence of the same pathologies excluding the coronary heart disease. Additionally, we assessed the heart rate at the time of thyrotoxicosis. It was defined as the average value, based on at least three measurements from the medical records. The analysis included only the values obtained during hyperthyroidism and before heart rate-reducing therapy administration.

Arterial hypertension was defined by the presence of essential or secondary hypertension history*.* This diagnosis was also made in case of antihypertensive medication use or if systolic blood pressure (SBP) of 140 mmHg or greater and/or diastolic blood pressure (DBP) of 90 mmHg or greater were found at least twice in a medical record. Hypertensive patients were divided into those with target ABP and those with above target ABP. The separation was made in accordance with ABP level, having been present most of the time.

Coronary heart disease was defined as a history of angina pectoris and/or myocardial infarction and/or recorded on electrocardiogram (ECG)/during Holter ECG monitoring silent myocardial ischemia and/or coronary angioplasty and/or coronary bypass.

Participants were categorized as having any rhythm disorder if it was present in diagnosis or registered on ECG/Holter ECG monitoring.

Heart failure was diagnosed based on the clinical criteria from the ESC guidelines, 2016 [25].

The metabolic parameters, widely known to be contributing to TAF development, such as body mass index, carbohydrate metabolism disorders and lipid profile were assessed. Body mass index was calculated by dividing weight in kilograms (kg) by height in metres squared (m2). The diagnosis of diabetes was established in case of a history of diabetes or antidiabetic medication use or if fasting blood glucose was 7 mmol/l or greater at least twice.

Moreover, smoking status was examined. Those who had been smoking before or during hyperthyroidism were classified as smokers. In TAF patients, smoking status was assessed before AF development.

We additionally analyzed potassium, hemoglobin and serum creatinine blood tests. An estimated glomerular filtration rate (GFR) was calculated with the CKD-EPI formula [26]. The potassium was assessed as both its increase and decrease can lead to cardiac arrhythmias, including atrial fibrillation. The hemoglobin level was assessed since anemia could cause myocyte dysfunction as a result of oxygen deprivation. The renal function was estimated, because renal failure had been shown predispose to TAF [12].

### Statistical analysis

Initially, 36 studied features were compared between patients with and without TAF by classical statistical methods*.* After that, we trained several intermediate prediction models with eight machine learning algorithms and selected the most important variables for inclusion in the final model. Then, the best performing optimal model was tested. Lastly, we ranked TAF predictors elicited from the optimal final model with the machine learning tehniques.

#### The initial analysis of the data: descriptive statistics and data exploration

The initial analysis was conducted by SPSS Statistics 17.0. All study features but TSH level were compared between those who developed TAF and those who did not. As TSH level occurred to be lower than the detection threshold in the majority of cases, it was excluded from the analysis. The normality of the distribution was checked by the Kolmogorov-Smirnov test. The various tests according to the distribution of variables and their characteristics were applied to evaluate the differences in the studied parameters: Mann-Whitney U test, Pearson’s chi-square formula and Fisher’s exact test. The *p*-value below 0.05 was assumed as statistically significant.

The data are presented as a mean ± standard deviation for abnormal distribution and as a median (interquartile range (IQR)) for abnormal distribution.

#### Derivation of a thyrotoxic atrial fibrillation prediction model

We used machine learning techniques and Python 3.6 for a TAF prediction model development.

Hereafter we described the steps of the model development.

##### Input variables

The analysis of previously examined TAF risk factors [4, 5, 7–19] and non-thyrotoxic AF prediction tools [24, 27–30] helped to define input variables for our models. First, we built several intermediate models, including more than 30 variables. Following that, to facilitate implementation of the model in clinical practice, we reduced the number of the predictors. We eliminated the features of low clinical effectuality such as serum potassium and lipids, since their concentrations are highly variable and strongly depend on the drugs taken and the diet. Then we removed the features of low importance for model output using multivariable statistical analyses. This analysis was based on the feature importance in decision trees method. Each decision tree included nodes and edges. For each node one feature was used for dividing observations into classes. Feature for this operation was chosen using some criteria, for classification tasks it was the Ginny coefficient, for regression tasks it was a variance of the feature. We calculated the influence on reducing the Ginny coefficient by each feature in average, this value was the feature importance indicator. As a result, ten most important and clinically feasible features were selected for the final model.

##### Preprocessing of the data

Preprocessing of the data comprised the following steps: normalization (module sklearn-preprocessing-normalize), scaling (module sklearn-preprocessing-scale), resampling for the balance of classes, replacing the data gaps.

##### Splitting the data

To evaluate the models’ quality, we randomly divided the study sample into two parts: 70% (*n* = 294) were used for the estimation of the models (training) and 30% (*n* = 126) for the validation (testing).

##### Used classification machine learning algorithms

We investigated the performance of the following machine learning methods: logistic regression, decision tree classifier, random forest classifier, dummy classifier, K-neighbors classifier, Bernoulli naive Bayes classifier, eXtreme Gradient Boosting classifier (XGB classifier) and Support Vector Machines for Classification.

##### Model performance assessment

The next step was to estimate the models’ performance. For this purpose, a five-fold cross-validation was performed. Quality indicators included accuracy, recall, precision, F1 score and area under the receiver operator characteristics curve (AUROC). The quantitative metrics of accuracy and AUROC are used for the classifier overall performance evaluation. Accuracy is a measure related to the total number of correct predictions from all predictions made. AUROC is a measure of the model’s performance which is based on the receiver operator characteristics curve that plots the tradeoffs between sensitivity and 1-specificity [31]. Precision is the number of true positives divided by the number of true positives and false positives. Recall (sensitivity) is the number of true positives divided by the number of true positives and false negatives. The F1 Score is the 2*((precision*recall)/(precision+recall)). According to these indicators, the best performing models were chosen. For these models hyperparameters were selected by a grid search method. Finally, we validated the best model with only the test set.

##### Model interpretation

To represent the prediction model graphically, three interpretability techniques (Feature importance, SHapley Additive exPlanations (SHAP) method and Partial dependence plot) were applied. Next, we will list and explain each of them.

1. Feature importance. To show the impact degree of each feature on the model output we used the charts, demonstrating the feature importance ranking. Feature importance is defined as the increase in the model’s prediction error after the values of the features were permuted. A feature is considered important if permuting its values increases the error [32].
2. SHAP or Shapley values method. The average contribution of each feature to the model prediction in different coalitions can be presented with SHAP plot. SHAP method is a solution concept of fairly distributing both gains and costs to several players working in coalition used in game theory [32].
3. Partial dependence plot. It shows the marginal effect one or two features have on the predicted outcome of a machine learning model [33]. To construct partial dependence plot, a variable is selected, and its value is continuously changing, whilst a change in the prediction value is observed and recorded.

#### Investigation of the TAF predictors elicited from the model

We used feature importance and SHAP values methods to rank and select the most important TAF predictors elicited from the model.

## Results

### Characteristics of the study group

The study cohort consisted of 420 subjects with a history of overt hyperthyroidism, 79.3% women and 20.7% men, whose mean age at the onset of hyperthyroidism was 44.3 ± 12.1 years. 94% of patients had GD, others had nonimmune thyroid pathology: TA or MNG. Detailed characteristic of the study population is shown in Table 2.

**Table 2:** Clinical characteristics of the participants: in the full cohort, AF and non-AF groups

Variable	Category	Full cohort ***N*** = 420	AF patients ***N*** = 127	Non-AF patients ***N*** = 293	***P***-value
**Demographic parameters**					
Sex, % (n)	Male	20.7 (87)	32.3 (41)	15.7 (46)	< 0.001
Age, years^a^		44.3 ± 12.1	48.9 ± 12.2	42.3 ± 11.5	< 0.001
**Characteristics of hyperthyroidism course**					
Hyperthyroidism duration, months^b^		10 (6;20)	18 (8;32)	8 (5.5;14.0)	< 0.001
Subclinical hyperthyroidism duration, % (n)	< 1 year	34.3 (128)	25.0 (29)	38.5 (99)	0.011
	≥1 year	65.7 (245)	75.0 (87)	61.5 (158)	
Number of relapses, % (n)	0	36.6 (140)	27.8 (30)	40.1 (110)	< 0.001
	1	33.8 (129)	26.9 (29)	36.5 (100)	
	≥2	29.6 (113)	45.4	23.4 (64)	
Hyperthyroidism origin, % (n)	Graves’ disease	94 (395)	89.8 (114)	95.9 (281)	0.015
	TA or MTG	6 (25)	10.2 (13)	4.1 (12)	
TSH, μIU/l^b^		< 0.01 (0.005;0.03)	< 0.006 (0.002;0.01)	< 0.01 (0.005;0.039)	–
fТ3, times above ULN range^b^		2.0 (1.5;3.0)	2.0 (1.4;2.80)	2.0 (1.5;3.1)	0.496
fТ4, times above ULN range^b^		1.8 (1.4;2.6)	1.8 (1.5;2.75)	1.8 (1.4;2.6)	0.473
Weight loss in the onset of hyperthyroidism, kg^b^		6 (0.0;12.0)	8 (0;16)	5.0 (0.4;10.0)	0.225
Extrathyroidal Graves ‘disease manifestations, % (n)^c^	ophthalmopathy	48.8 (191)	44.2 (50)	50.7 (141)	0.489
	pretibial myxedema	1.0 (4)	0.9 (1)	1.1 (3)	
TSH receptors antibodies, times above ULN range ^c^		8.4 (3.6;26.7)	9.9 (4.5;29.7)	9.3 (4.1;28.1)	0.716
**Metabolic parameters, smoking status and blood tests during hyperthyroidism**					
Body mass index, kg/m2^b^		25.4 (22.4;30.0)	26.9 (23.6;30.5)	24.7 (21.9;29)	0.002
Overweight, % (n)		27.7 (108)	24.5 (66)	34.7 (42)	0.002
Obesity, % (n)	level 1	16.9 (66)	17.5 (47)	15.7 (19)	
	level 2	6.4 (25)	4.1 (11)	11.6 (14)	
	level 3	1.5 (6)	1.1 (3)	2.5 (3)	
Carbohydrate metabolism disorders, % (n)	IFG	4.4 (15)	4.1 (5)	4.5 (10)	0.460
	IGT	2.6 (9)	2.4 (3)	2.7 (6)	
	DM (type 1 or 2)	9.6 (33)	13 (16)	7.7 (17)	
Lipid profile^b^	TC, mmol/l	4.2 (3.5; 5.2)	4.2 (3.5;5.2)	4.1 (3.5;5.2)	0.824
	TG, mmol/l	1.0 (0.8; 1.4)	0.98 (0.8;1.2)	1.1 (0.8;1.5)	0.149
	LDL, mmol/l	2.2 (1.4; 3.1)	2.4 (1.5;3.5)	2.1 (1.3;2.9)	0.108
	HDL, mmol/l	1.1 (0.9; 1.4)	1.1 (0.9;1.5)	1.1 (0.9;1.4)	0.858
Smokers, % (n)		28.4 (113)	35.2 (44)	25.3 (69)	0.042
Plasma creatinine level, μmol/l^a^		61.2 ± 17.8	65.5 ± 22.6^a^	58.7 ± 13.9^a^	0.017
GFR, ml/min/1.73 m2^b^		104.6 (85.0; 125.1)	101.9 (82.7;123.3)	105.9 (87.1;127.2)	0.146
GFR 60–90 ml/min/1.73 m2, % (n)		25.9 (56)	27.8 (22)	24.8 (34)	0.027
GFR < 60 ml/min/1.73 m2, % (n)		5.2 (11)	10.2 (8)	2.2 (3)	
Plasma potassium level, mmol/l^a^		4.4 ± 0.5	4.5 ± 0.6	4.4 ± 0.4	0.122
Hypokalaemia (potassium < 3.5 mmol/l), % (n)		2.8 (6)	5.1 (4)	1.5 (2)	0.126
Haemoglobin, g/l^a^		132 ± 17.8	132.1 ± 19.1	131.9 ± 17.1	0.907
Anemia (haemoglobin < 120 g/l for women, < 130 g/l for men), % (n)		22.1 (53)	27.6 (24)	19.0 (29)	0.121
**Initial cardiovascular status**					
Arterial hypertension, % (n)		30.1 (124)	45.6 (57)	23.3 (67)	< 0.001
Above target ABP, % from hypertensive patients (n)		42.7 (53)	45.6 (26)	40.3 (27)	0.588
Heart failure, % (n)		4.8 (20)	10.5 (13)	2.4 (7)	0.002
Coronary heart disease, % (n)		12.9 (54)	17.3 (22)	11.0 (32)	0.082
Rhythm disorders before hyperthyroidism, % (n)	PAC	0.5 (2)	0	0.7 (2)	0.249
	PVC	0.7 (3)	1.6 (2)	0.3 (1)	
**Cardiovascular status during hyperthyroidism**					
A rterial hypertension, % (n)		54.8 (228)	75.6 (93)	46.1 (135)	< 0.001
Above target ABP, % from hypertensive patients (n)		28.1 (64)	32.3 (30)	25.2 (34)	0.294
Congestive heart failure, % (n)		31.4 (97)	51.1 (46)	23.3 (51)	< 0.001
Heart rate during hyperthyroidism, bpm^b^		94 (85; 103.5)	96 (88;105)	92 (84;102)	0.181
Sinus tachycardia during hyperthyroidism, % (n)		64.3 (222)	73.6 (53)	61.9 (169)	0.073
Rhythm disorders during hyperthyroidism, % (n)	PAC	44.9 (140)	87.5 (49)	35.5 (91)	< 0.001
	PVC	16.2 (43)	50 (24)	8.8 (19)	< 0.001
	SVT	13.3 (34)	28.2 (11)	10.6 (23)	< 0.001
	VT	5.1 (13)	20.5 (8)	2.3 (5)	
	WAP	2 (5)	2.6 (1)	1.8 (4)	

*AF* atrial fibrillation; *TA* toxic adenoma; *MTG* multinodular toxic goiter; *TSH* thyroid-stimulating hormone; *fT3* free triiodothyronine; *fT4* free tetraiodothyronine; *ULN* upper limit of normal; *IFG* impaired fasting glucose; *IGT* impaired glucose tolerance; *DM* diabetes mellitus; *TC* total cholesterol; *TG* triglycerides; *LDL* low density lipoproteins; *HDL* high density lipoproteins; *GFR* glomerular filtration rate; *PAC* premature atrial contraction; *PVC* premature ventricular contraction; *ABP* arterial blood pressure; *SVT* supra-ventricular tachycardia; *VT* ventricular tachycardia; *WAP* wandering of atrial pacemaker

^a^ mean ± S.D.

^b^ median (interquartile range or percentiles 25; 75)

^c^ only in subjects with Graves’ disease

TSH level was lower than the detection limit of 0.01 μIU/l in the majority of cases. When calculating the median for the group, it was considered that these individuals had TSH level of 0.01 μIU/l. The median, thereby, was presented as < 0.014 μIU/l (Table 2).

The lipid panel assessment showed that TC, LDL and TG mean levels were target (for low or moderate cardiovascular risk). HDL mean level for the men and women was at the lower limit of the target range.

The proportion of diabetes cases was high due to the big amount of diabetes patients at Almazov centre and Pavlov University. They were enrolled in the study because they had hyperthyroidism as a secondary diagnosis.

Table 2 also shows cardiovascular status of the participants before and during hyperthyroidism. Before hyperthyroidism development, 30.1% of patients had arterial hypertension, 42.7% of which had above target ABP most of the time. During hyperthyroidism, the proportion of hypertensive patients significantly increased to 54.8%, but the participants were less likely to have above target ABP (28.1%). Similarly, the frequency of congestive heart failure dramatically increased after hyperthyroidism development from around one in twenty (4.8%) to more than one in four (31.4%). Coronary heart disease was detected in 12.9% of subjects, 31.5% of which had a prior history of myocardial infarction.

Heart rhythm disorders before hyperthyroidism were established in only 1.2% of participants. During hyperthyroidism 81.5% of participants were found to have dysrhythmias, the most common of which was premature atrial contraction (PAC) (44.9%). The median heart rate during hyperthyroidism of the study cohort was 94 bpm (IQR 85; 103.5 bpm). Sinus tachycardia (heart rate ≥ 90 bpm) was found in 64.3% of participants. Regarding TAF, we intentionally enrolled TAF subjects in the study cohort, which explains the abnormally high percentage (30.2%) of these patients in our sample.

### Differences in study variables between TAF and non-TAF patients

#### Demographic, metabolic parameters, smoking status, blood tests, characteristics of hyperthyroidism course

In TAF group we observed greater proportion of men, smokers, patients with nonimmune thyrotoxicosis, with prolonged duration (1 year and more) of subclinical hyperthyroidism and with multiple relapses (≥2) of hyperthyroidism than in non-TAF group. TAF individuals had elder age, higher body mass index, more prolonged hyperthyroidism duration and higher serum creatinine level compared to non-TAF patients (Table 2).

#### Cardiovascular status

Among individuals diagnosed with TAF, there were more cases of arterial hypertension and congestive heart failure, both before and during hyperthyroidism development, compared to non-TAF patients. In addition, there were more participants who had above target ABP most of the time in TAF group compared to non-TAF subjects. The data are shown in Table 2.

There was no statistically significant difference in the coronary heart disease frequency depending on the TAF presence.

Before hyperthyroidism there were too few cases of arrhythmias (1.2%, *n* = 5) to analyze its association with TAF. The analysis of the heart rhythm disorders during hyperthyroidism showed that TAF patients were more likely to have both atrial and ventricular premature contraction (PVC) than non-TAF subjects. The frequency of other arrhythmias, detected during hyperthyroidism, was also higher in TAF group (Table 2).

There was no association of TAF frequency with heart rate. The median heart rate for patients diagnosed with TAF was 96 bpm (IQR 88.3; 106 bpm), compared with 92 bpm (IQR 84; 102 bpm) for non-TAF individuals, but this difference was not statistically significant: *p* = 0.181. Similarly, the frequency of sinus tachycardia (heart rate 90 bpm or more) was higher among TAF patients, compared with non-TAF participants (73.6% vs 61.9%), but the difference still was not significant: *p* = 0.065.

#### Heart rate-reducing therapy

It should be noted that all patients before hyperthyroidism and 97% of those during hyperthyroidism received beta-blockers as heart rate-reducing therapy. Before hyperthyroidism, a larger proportion of patients in the TAF group was receiving heart rate-reducing therapy compared with non-TAF group: 13% vs 5.9%, *p* = 0.015. There was no significant difference between TAF and non-TAF participants on heart rate-reducing therapy during hyperthyroidism.

### Thyrotoxic atrial fibrillation prediction models

#### Derivation and validation of the prediction models

The final TAF prediction model included ten variables: age (1), sex (2), hyperthyroidism duration (3) and number of relapses (4), heart rate (5), the presence of arterial hypertension (6) and rhythm disturbances (PAC (7), PVC (8); supraventricular tachycardia, non-sustained ventricular tachycardia, wandering of atrial pacemaker (9)) and heart rate-reducing therapy (10). The last six features were evaluated during hyperthyroidism before TAF development.

According to the cross-validation method, among the eight machine learning methods, XGB classifier achieved the highest accuracy. The best performing XGB model was validated on the test set. The performance metrics for this model on the test set were as follows: 84% accuracy, 82% precision and 77% recall.

The model discrimination ability was estimated by the AUROC. The final XGB model achieved the high predictive capacity with AUROC of 0.93, when it was calculated with the full sample. The AUROC on the test set was slightly worse: 0.89.

#### Interpretation of the prediction models

In this section we present the results of applying three interpretability techniques for our TAF prediction model. They are as follows: Feature Importance, Shapley Values and Partial Dependence Plot.

##### Feature importance method

**Fig. 1:**
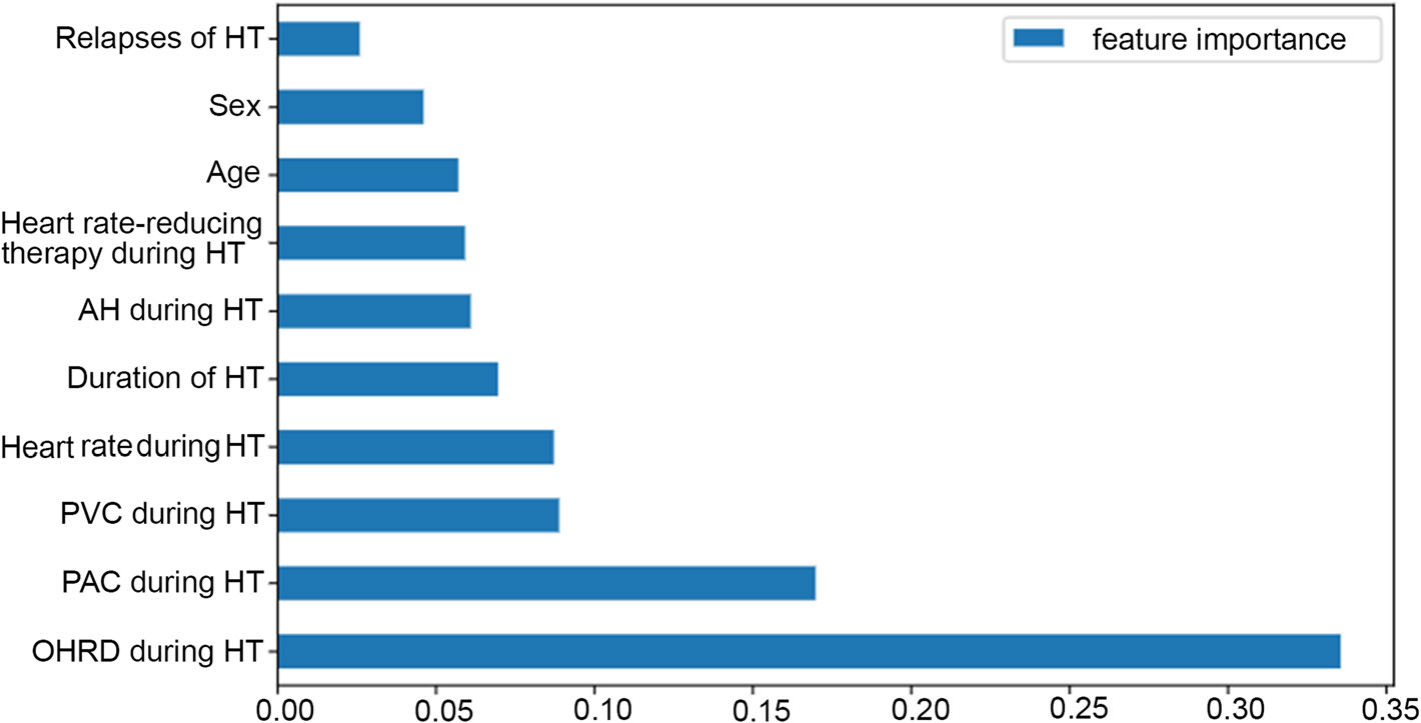
Feature importance in predicting thyrotoxic atrial fibrillation according to the developed model. HT = hyperthyroidism. AH = arterial hypertension. PVC = premature ventricular contraction. PAC = premature atrial contraction. OHRD = other heart rhythm disorders

Figure 1 shows the ranking of the input features importance. As shown in the figure, the feature other heart rhythm disorders during hyperthyroidism is the most important one, followed by PAC and PVC during hyperthyroidism. The variable relapses of hyperthyroidism is the least significant feature.

##### Shapley values (SHAP method)

**Fig. 2:**
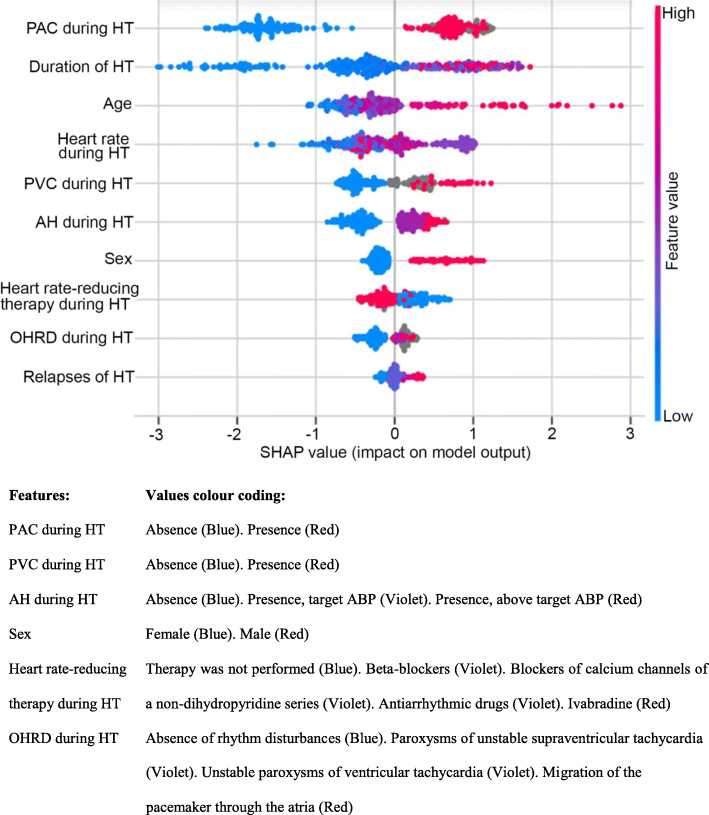
Shapley values of thyrotoxic atrial fibrillation predictors inferred from the final model. HT = hyperthyroidism. AH = arterial hypertension. PVC = premature ventricular contraction. PAC = premature atrial contraction. OHRD = other heart rhythm disorders

Figure 2 shows the Shapley values for the model’s input features. The figure is organized in descending order of the feature importance, so that the PAC during hyperthyroidism contributes most to the TAF prediction. The figure also shows the feature values increasing and reducing TAF risk. The advanced age and long duration of hyperthyroidism have the highest positive impact on TAF risk (raised the risk), whereas short duration of hyperthyroidism, absence of PAC and low heart rate during hyperthyroidism have a highest negative impact on TAF risk (reduced the risk).

**Fig. 3:**
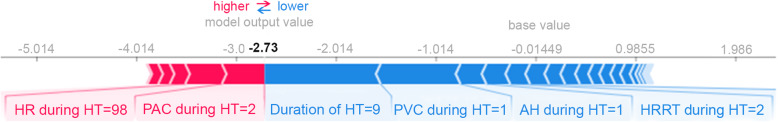
Example of working model. HR = heart rate. HT = hyperthyroidism. PAC = premature atrial contraction. PVC = premature ventricular contraction. AH = arterial hypertension. HRRT = heart rate-reducing therapy

Figure 3 provides the interpretation of the model prediction for one random patient. We highlighted the variables that had a strong impact on the model prediction for the patient. The influence values of the features were calculated by the SHAP method. Features increasing TAF probability were marked in red, the ones reducing TAF **-** in blue. Heart rate during hyperthyroidism of 98 bpm and PAC during hyperthyroidism increased the probability of TAF most strongly. Features, reducing the probability of TAF for this particular patient, were as follows: short duration of hyperthyroidism (Duration of HT = 9), absence of PVC (PVC during HT = 1), absence of arterialhypertension during hyperthyroidism (AH during HT = 1) and heart rate-reducing therapy during hyperthyroidism (HRRT during HT = 2). The duration of hyperthyroidism had the strongest absolute influence on the resulting value. As a result, TAF development probability of 7% was calculated for this patient.

##### Partial dependence plot method

**Fig. 4:**
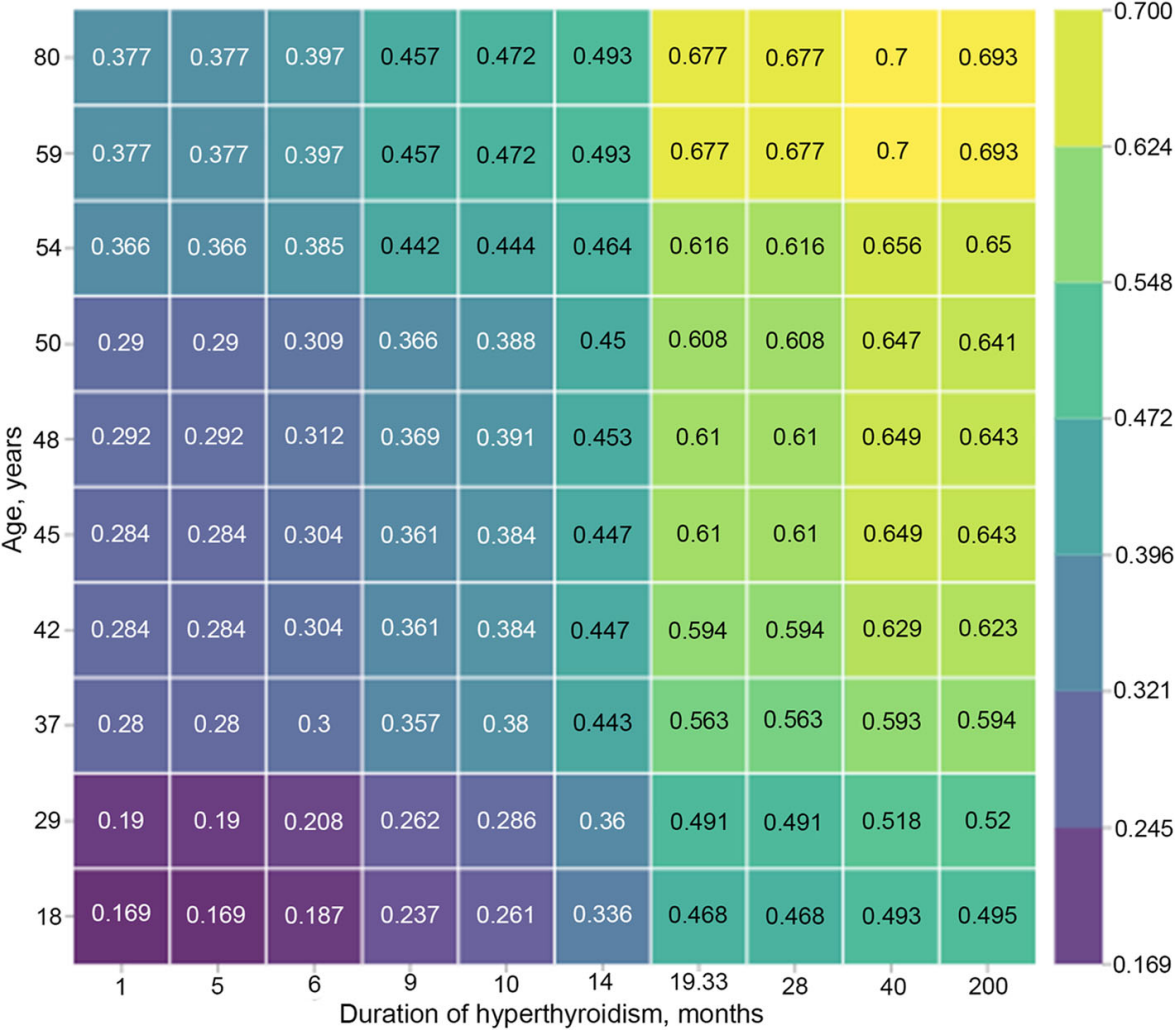
Partial Dependence plot for age and duration of hyperthyroidism

Figure 4 shows the cumulative effect of two predictors. This effect was calculated by the Partial dependence plot method. The scale shows how age and hyperthyroidism duration values alterations change TAF probability, provided the other features values are fixed. If a patient was older than 33, and hyperthyroidism duration was more than 20 months, the patient had TAF development risk more than 0.5. These two features increased the probability of TAF, when their values were increasing. Minimal risk value was 0.16 for patients who were younger than 20 with the short period of hyperthyroidism. Maximal risk value was 0.7 for patients who were older than 60 with the period of hyperthyroidism for over 40 months.

### Top thyrotoxic atrial fibrillation risk factors elicited from the prediction model

The next aim of the study was to rank TAF predictors by the importance value and identify the most important features. For this purpose, we used feature importance (Fig. 1) and Shapley values (Fig. 2) techniques, assessing the features impact on the model output in two different ways. If consider the top five features, the four of them are the same in both methods. They are as follows: hyperthyroidism duration, PAC, PVC and heart rate during hyperthyroidism. According to the feature importance method, the five most important factors also include different rhythm disorders during hyperthyroidism, estimated collectively (Fig. 1). By contrast, according to the SHAP method the top five features include age.

When creating a list of five most important TAF risk factors, we took into account all the results of both methods. Apart from the four consistent predictors, we included age for three reasons listed below. Firstly, it is more difficult to obtain information about rhythm disorders than about age. Data collection challenges may provoke some errors. Secondly, there were many missing values for the variable other rhythm disorders during hyperthyroidism by contrast to age, for which there was none. Finally, age is an acknowledged TAF risk factor [4, 5, 7–14]. Thus, the top five TAF predictors, elicited from our model, include age, hyperthyroidism duration, PAC, PVC and heart rate during hyperthyroidism.

## Discussion

High TAF prevalence among hyperthyroid patients [4, 34–36] and the lack of any TAF prediction system motivated this research. To the best of our knowledge, we developed the first TAF prediction model. Top five risk factors emerging from our model include age, hyperthyroidism duration, PAC, PVC and heart rate during hyperthyroidism.

We believe a TAF prediction tool would be of great use. It is an indispensable tool for the early identification of individuals with high risk of TAF. It would give practitioners the resources to determine indications for more intensive medical care or early radical treatment of hyperthyroidism (total thyroidectomy, radioiodine therapy) [37–39]. This will ultimately lead to a decrease in TAF frequency. The practical implications of the current study have been TAF prevention, and, as a result, decrease in health-care costs.

We used machine learning methods to build the model. Among eight evaluated machine learning classifiers, XGB classifier achieved the best performance metrics. Our final XGB model had a reasonable accuracy of 84% and good discrimination ability with AUROC of 0.89 on the test set. Among 36 investigated potential TAF predictors, ten were selected as input variables for the model. The variables were ranked by feature importance and SHAP methods (Figs. 1 and 2). These methods calculate the importance value in different ways and, therefore, could produce differing results [32]. The prediction model takes into account variables characteristics displayed by both methods. Hereafter, we will discuss the predictors inferred from the model in comparison with the previous findings in the field.

To begin with, we will consider our findings on rhythm disorders as TAF predictors. Both feature importance and SHAP methods showed that PAC and PVC during hyperthyroidism are among five most important TAF risk factors (Figs. 1 and 2). It seems that PAC and PVC impact on TAF had not been investigated before, and, in our study, they were defined as novel TAF predictors.

We would like to emphasize that our model confirms such widely acknowledged TAF risk factors as age, sex and hyperthyroidism duration. According to the SHAP method, hyperthyroidism duration had the highest impact on model output*,* while age and sex ranked the fourth and the seventh out of ten factors, respectively (Fig. 2). In contrast, the feature importance method shows that hyperthyroidism duration had the mean importance value among ten input variables. Age and sex were almost the least important factors (Table 1).

The next known TAF risk factor is heart rate. Earlier, heart rate above 80 bpm was mentioned as a TAF predictor [12]. However, our findings were dissimilar. Machine learning methods showed the nonlinear interaction between heart rate and TAF. Figure 2 shows that low heart rate reduces TAF risk, the medium values mostly increase it, but the highest ones have a minimal impact on model output. The latter phenomenon could be due to the scarce information obtained. We had only several heart rate measurements from medical records, which may not reflect the actual heart rate.

The concomitant cardiovascular diseases is another TAF predictor. As early as in 1959 G. Sandler and G.M. Wilson showed that TAF frequency was significantly higher in patients with cardiovascular diseases preceding hyperthyroidism [15]. According to the more recent studies, coronary heart disease, congestive heart failure and high blood pressure significantly increase TAF risk [4, 7, 12, 17]. Our findings on cardiovascular diseases were mixed. On the one hand, we showed that hypertension and congestive heart failure existence (both before and during hyperthyroidism) raise TAF risk. Moreover, arterial hypertension during hyperthyroidism was the only sufficiently important variable to be included in the model. On the other hand, contrary to the majority of studies [4, 12, 17], we did not find coronary heart disease or history of myocardial infarction to predict TAF.

Next, we would like to consider the less investigated TAF predictor, that is, heart rate lowering drug use. It is worth noting, that we explored the heart rate-reducing therapy both before and during hyperthyroidism as two separate variables. All patients before hyperthyroidism and 97% of those during hyperthyroidism received beta-blockers as this therapy. The heart rate-reducing therapy before hyperthyroidism had a minimal impact on TAF prediction according to machine learning methods and, based on that, was excluded from the prediction model. It might be of interest to note, that the classical statistical methods showed that the patients receiving beta-blockers before hyperthyroidism were more prone to TAF. However, there is some evidence that beta-blockers could decrease TAF incidence [12, 18]. The divergent results could be explained by the following fact. In our study almost all the participants who received beta-blockers before hyperthyroidism had concomitant cardiovascular diseases. All of them had arterial hypertension and 78.8% - coronary heart disease. These cardiovascular diseases are known to significantly contribute to TAF. The heart rate-reducing therapy during hyperthyroidism was included in the model. We found this therapy to decrease TAF risk. Therefore, beta-blockers use during hyperthyroidism might be an effective TAF preventive measure.

Lastly, we consider the number of hyperthyroidism relapses, which was not previously mentioned in the literature as a TAF predictor. It was the least important variable of the model (Figs. 1 and 2); therefor we did not single out it as a new TAF risk factor.

The study comes with some limitations. Firstly, being retrospective, the research has no randomization factor that cuts off unknown or unrecorded effects on the studied features. Secondly, the sample size is smaller than the optimal one for machine learning methods. In addition, the study participants were recruited from two healthcare organizations. Consequently, the model’s accuracy may change when tested in different cohorts. For this reason, the tool needs to be validated in other studies. As for the new TAF predictors, PAC and PVC, since there are no other studies testing their predictive value, these results also need to be confirmed*.* Another limitation is the fact, that three input variables (PAC, PVC and other rhythm disorders during hyperthyroidism) required ECG results. These variables complicate the collection of information, necessary for TAF risk calculation. On the other hand, the absence of echocardiographic parameters assessing left ventricular function among studied parameters is a limitation of the study. Left ventricular dysfunction is an important determinant of prognosis in patients with previous myocardial infarction and heart failure, which were enrolled in this study. And despite inclusion of echocardiographic parameters to the prediction model would complicate the use of the model in practice, it most likely would increase the models’ prediction quality. The next limitation regards gathering information on rhythm disorders. Holter monitoring, performed in the onset of hyperthyroidism, would be the most appropriate method for rhythm disorders detection. In our study, the data were ascertained either from ECG and Holter monitoring, or from the anamneses and diagnoses in the medical records. Another limitation is the absence of TAF predictors cut-off values, after which TAF risk dramatically increases. It is important for the prediction instrument use in practice. New prospective and adequately powered studies are required to identify which TAF predictors thresholds pose patients with hyperthyroidism at an exceedingly high risk to develop TAF. These specific thresholds may prompt dedicated treatment options. Having determined TAF predictors cut-off values, TAF predictive scale may be created, which will be convenient to calculate AF risk in patients with thyrotoxicosis. Finally, our prediction model has been developed without determination of the period the forecast is intended to cover. This makes the model less convenient for practical use, because preventive measures for definite period are more effective.

## Conclusions

We have developed the machine learning model which predicts TAF with 84% accuracy. It seems to be the first available TAF prediction tool.

In addition, we have identified that TAF risk factors with the highest predictive ability include PAC, PVC, age, heart rate during hyperthyroidism and hyperthyroidism duration. All listed above arrhythmias seem to be the new TAF predictors. Further studies have to confirm these new TAF risk factors, as well as validate the usefulness and appropriateness of our model in independent cohorts. The study could serve as a basis for further research focused on TAF prediction improvement and facilitation of thyrotoxic patients’ management. Our results could be considered in the development of TAF risk scales, introduction of which into the clinical practice has a potential to reduce TAF incidence.

## Data Availability

The datasets analysed during the current study are available from the corresponding author on reasonable request.

## Abbreviations

ABP: Arterial blood pressure
AF: Atrial fibrillation
AUROC: Area under the receiver operator characteristics curve
bpm: Beat per minute
CKD-EPI: Chronic Kidney Disease Epidemiology Collaboration
DBP: Diastolic blood pressure
ECG: Electrocardiogram
ESC: European Society of Cardiology
fT4: Free tetraiodothyronine
fT3: Free triiodothyronine
GD: Graves’ disease
GFR: Glomerular filtration rate
HDL: High density lipoproteins
IQR: Inter quartile range
LDL: Low density lipoproteins
MTG: Multinodular toxic goiter
PAC: Premature atrial contraction
PVC: Premature ventricular contraction
SBP: Systolic blood ressure
SHAP: SHapley Additive exPlanations
TA: Toxic adenoma
TAF: Thyrotoxic atrial fibrillation
TC: Total cholesterol
TG: Triglycerides
TSH: Thyroid-stimulating hormone
ULN: Upper limit of normal
XGB classifier: eXtreme Gradient Boosting classifier
